# The Experiences of Intermediate‐Titled Clinical Nurses’ Promotion to Senior Titles: A Phenomenological Study

**DOI:** 10.1155/jonm/1994161

**Published:** 2026-06-23

**Authors:** Sufen Zheng, Haixiang Zhu, Jiali Gong

**Affiliations:** ^1^ Nursing Department, Sir Run Run Shaw Hospital, Zhejiang University School of Medicine, Hangzhou, 310016, Zhejiang, China, zju.edu.cn

**Keywords:** career advancement, intermediate nurses, phenomenological study, promotion, senior titles

## Abstract

**Background:**

Nurses show low enthusiasm for senior title promotion despite its career benefits. Understanding their experiences and difficulties can inform more effective policy implementation.

**Introduction:**

The nurse promotion system directly affects remuneration and work enthusiasm. Mobilizing nurses’ initiative within this framework remains a key challenge for policymakers and hospital administrators.

**Aim:**

To explore the views and experiences of clinical nurses with intermediate titles on the promotion system of senior titles.

**Methods:**

A descriptive phenomenological study was conducted. A total of 13 clinical nurses with intermediate professional titles from 11 different departments (the average years of obtaining the intermediate title were 7.54 years) participated in face‐to‐face semistructured interviews. The data were analyzed using Colaizzi’s seven‐step data analysis method (1978). The COREQ guidelines were followed for reporting. Rigor was ensured through member checking and peer debriefing.

**Results:**

Five categories emerged: Escape (perceived inadequacy, clinical/family burden, and lack of guidance/role models); Transform (clinical influence, environmental impact, and enhanced self‐awareness); Challenge (research learning, degree hurdles, and trial/failure); Reflection (early career planning, perseverance, and continuous self‐growth); and Expect (career/psychological support, research environment, and diversified criteria). These categories formed a cyclical process from Escape to Transform, interrupted by Challenge, leading to Reflection, and culminating in Expect for systemic change.

**Discussion:**

Nursing managers and educators should attach great importance to the promotion experience of nurses, promptly address their promotion needs, and provide career planning training and guidance as early as possible.

**Conclusions:**

By planning career development in advance, actively learning and enhancing professional and research capabilities, the comprehensive ability and professional pride of nurses can be strengthened, and the promotion rate of senior professional titles for nurses can be increased.

**Implications for Nursing Policy:**

Nurse managers, policy makers, and nursing schools should jointly design a career development framework including mentorship, protected research time, methodological support, career planning workshops, diversified criteria, and innovation‐friendly environments. Context‐tailored implementation may enhance nurses’ promotion enthusiasm and success rates.

## 1. Introduction

The advancement of nurses’ professional titles constitutes the pivotal pathway for their professional development and serves as a crucial component in the construction of the nursing talent workforce [1, 2]. Current research indicates that nurses encounter various challenges during the promotion process. The success rate of promotion from intermediate to senior positions is low, but the reasons for this have not been further explored [1, 3]. In the Chinese context, nurses holding intermediate titles must satisfy stringent evaluation criteria to be promoted to senior titles. Such criteria include the publication of research papers, fulfillment of continuing education hours, and accumulation of clinical work experience [4]. Concurrently, this progression is frequently accompanied by substantial occupational stress and distinct psychological experiences [2, 5]. Furthermore, delving deeper into this psychological experience can provide valuable feedback and suggestions for the implementation of the promotion system.

From an international perspective, nursing career advancement pathways differ markedly across healthcare systems. In many Western countries, advancement to senior or advanced nursing roles is predominantly competency‐based, emphasizing clinical expertise, role expansion, and professional autonomy, rather than mandatory research productivity. Take the United States as an example, as the birthplace of the nurse promotion system, it assesses nurses’ capabilities through a hierarchical advancement model [6]. In 1982, Benner first proposed the “Novice to Expert” theoretical model [7], suggesting that the development of a nurse’s comprehensive ability requires going through five stages. This theory serves as the theoretical basis for progressive advancement and has now been widely applied in clinical settings [8, 9]. However, in Chinese, the application of such developmental frameworks is often overlaid with stringent research demands, which may disrupt the natural progression envisioned by Benner’s model. International academic research has shown that nurses typically undergo a “role transition” during their career development, which requires them to maintain a balance between clinical practice and academic pursuits [10]. In contrast, despite recent national reforms in China advocating for practice‐oriented and capability‐based evaluation, the promotion trajectory for nurses—particularly from intermediate to senior titles—continues to place substantial emphasis on research‐related indicators in practice. National policy documents, including the Guiding Opinions on Deepening the Reform of the Professional Title System for Health Professionals [11], explicitly propose breaking away from the “paper‐only” evaluation model and encourage the use of clinical cases, nursing practices, and representative professional achievements as key assessment criteria. However, at the institutional level, research output and academic credentials remain prominent benchmarks in promotion processes, especially in tertiary research hospitals, which may intensify the pressure experienced by clinical nurses [12, 13]. This gap between national policy intentions and institutional implementation is a distinctly Chinese phenomenon that shapes the lived experiences of the nurses in our study. For instance, the 2023 regulations in Zhejiang Province stipulate that applicants for senior professional titles are expected to demonstrate research leadership or publication capacity, thereby posing considerable challenges for nurses whose primary responsibilities remain patient care [14]. Such provincial requirements exemplify how local implementation of national policies can inadvertently maintain research‐centric criteria, a tension less commonly observed in Western competency‐based systems. Moreover, nurses with extensive seniority often confront the phenomenon of “career plateauing,” characterized by promotion bottlenecks and diminished self‐efficacy [15]. Intermediate‐title nurses are situated in a particularly sensitive position, as they are simultaneously required to meet high clinical workload demands and respond to evolving policy expectations regarding research and professional development, while nurses with less seniority are hampered by a lack of diversified cultural competencies [16]. In conclusion, in China, the improvement of research capabilities remains the primary requirement. This has brought about completely different pressures for clinical nurses and also affected their career development.

Under the imperatives of the new round of medical reform, the optimization of the human resource incentive mechanism within public hospitals has emerged as a critical area of reform. As an integral part of clinical medical care, nursing staff, as frontline healthcare providers, are indispensable to the medical team and play a vital role in hospital development. For an extended period, clinical nurses have endured significant work pressure and high‐intensity workloads, while their remuneration often fails to adequately reflect the value of their labor. Within this policy context of promoting high‐quality development and talent‐centered evaluation, understanding how intermediate‐title nurses perceive and experience the promotion system becomes essential for assessing the alignment between policy intentions and frontline realities. On the other hand, the challenge of fully stimulating nurses’ enthusiasm and initiative within the framework of the incentive mechanism reform remains a pressing issue for policymakers and hospital administrators alike.

## 2. Background

In China, the nurse professional title system comprises junior, intermediate, and senior titles—the latter subdivided into associate and full senior. Promotion to senior titles requires a combination of examination and evaluation. National policy mandates that associate senior candidates hold a bachelor’s degree or higher, have at least 5 years of experience as a supervisor nurse, and demonstrate both research achievements (e.g., papers and projects) and excellent clinical performance [4]. In reality, nurses’ promotion experiences are complex and diverse. Firstly, research pressure has emerged as a significant obstacle. Surveys have indicated that nurses in tertiary hospitals struggle to balance research with their heavy clinical workloads, leading to a decline in their willingness to pursue promotion [5, 17]. Secondly, policy disparities exacerbate regional imbalances. For instance, in Zhejiang Province, “cross‐level competition” is permitted. That is, those who have already obtained other professional titles in a certain category can, under certain conditions, be reclassified as the same level of health professional title. This to some extent increases the difficulty for nurses to advance to higher professional titles [14]. However, most provinces still follow the model of step‐by‐step promotion. Additionally, primary hospitals often have limited resources, making it more challenging for nurses in these institutions to access training opportunities [18]. Moreover, professional identity and organizational support have a substantial impact on promotion experiences. Senior nurses frequently opt for early retirement because “their career value is not fully recognized” [12], while young nurses may feel confused due to the lack of career‐planning guidance [19]. It is also worth noting that the promotion cycle for nurses in China is longer compared to international standards (for example, the promotion of advanced practice registered nurses (APRN) in the United States requires a master’s degree and clinical assessment). Furthermore, there is a lack of flexible certification pathways for specialized nurses [3], which further increases the uncertainty in career development. It can be seen that, unlike the junior nurses who focus solely on clinical skills learning, and unlike the senior nurses who have already attained the highest professional title, the position of intermediate‐level nurses is similar to that of a “sandwich”; they are simultaneously under multiple pressures from clinical workload, family responsibilities, and research requirements. They also face the highest risk of career stagnation [20], as at this stage, the gap between effort and promotion outcomes is the most significant. Moreover, they are the main group where there is a discrepancy between policy and practice; the country’s reform advocates for practice‐based assessment [11], but research‐oriented hospitals still prioritize research outcomes.

In summary, career promotion is a crucial stage for nurses, as it affects their career development and, in turn, the quality of medical care. Although there have been studies exploring the influencing factors of nurses’ career development [21, 22], there are still relatively few qualitative studies on the specific experiences and feelings of Chinese nurses with intermediate professional titles during their promotion to senior professional titles. As both a philosophy and a research method, descriptive phenomenology seeks to describe the essence of lived experiences [23], promote the understanding of people, and explain the nature of the action. Therefore, the aim of this study is to explore the experiences and feelings of nurses during the process of advancing from intermediate to senior professional titles and to conduct an in‐depth analysis of the real‐life experiences of this group. Based on these findings, the study aims to optimize the professional title evaluation policies, enhance the career satisfaction of nurses, and thereby improve the quality of medical care.

## 3. Methods

The consolidated criteria for reporting qualitative research (COREQ) checklist was used to guide the reporting of this qualitative research [24].

### 3.1. Design

A descriptive phenomenological design was employed to explore clinical nurses’ experiences and emotions during senior title promotion. This approach aims to capture the essence of lived experiences [23] without embellishment, thereby helping to explain the low proportion of nurses advancing to senior titles. Two key concepts underpin this method: intentionality, the idea that thoughts and actions are oriented toward experiences [23], justifying open participant expression, and phenomenological reduction (bracketing), requiring researchers to set aside prior knowledge and biases to describe experiences in a pure, universal sense [25].

### 3.2. Setting

This study was conducted in the affiliated hospital of a research university. The hospital has over 3000 nurses, and more than 1500 of them hold intermediate titles, which accounts for more than half. The next promotion target for nurses with intermediate titles is the senior title. The reasons for choosing this hospital are as follows: (1) it is one of the research hospitals with a strong atmosphere of scientific research and innovation, setting a precedent among the top‐three hospitals in Zhejiang Province. (2) The researcher works in the hospital, engaging in work related to nursing teaching and management, which provides certain advantages in the selection of sample diversity.

### 3.3. Sampling and Participants

This study employed the purposive sampling method, taking into account nurses from diverse specialties and departments, as well as their gender, marital status, and reproductive status. Purposive sampling enables the selection of participants who possess in‐depth knowledge and rich experience regarding the phenomena under investigation [26]. The inclusion criteria were as follows: (1) clinical nurses in a comprehensive grade‐three hospital. (2) Promotion to intermediate for 5 years or more. (3) No communication disorder or no mental disease. (4) Voluntarily participation in the study with a signed informed consent form, including willing to participate in the final validation phase of data analysis. The exclusion criteria were as follows: (1) leave for various reasons in the past 5 years for more than 1 year. (2) Have the intention to resign. (3) Vulnerable groups in the research unit, such as having a superior and subordinate relationship with the researcher. Exit criteria: respondents wished to withdraw for various reasons, such as being no longer able to participate or unwilling to participate in the study process.

With the approval of the nursing department, the researcher obtained the basic information list of the hospital’s chief nurses for 5 years or more. According to the purpose sampling method, the sample size required for qualitative research was considered to be small. Initially, the researcher selected 20 candidate interviewees from the list according to the principle of maximum difference and called them to inform the purpose of the study and the data collection procedure. They are also informed that participation in this study is voluntary and that they can withdraw at any time in the process without any consequences, without affecting their work and promotion, and without any impact on their work if they do not participate. If the interview candidate is willing to participate, a one‐to‐one WeChat contact will be established with the researcher, and the time and place of the interview will be determined through WeChat contact. In the end, five did not accept the invitation; one person withdrew from the study before the interview was finished because of a temporary family affair; one participant did not participate in the study due to data saturation. Finally, 13 nurses participated in this study, and the basic information is shown in Table 1.

**TABLE 1:** Study population characteristics.

*N* = 13	No. (%)
Gender	
Female	10 (77%)
Male	3 (23%)
Age	
< 35 years	5 (38%)
≥ 35 years	8 (62%)
Education background	
Junior college	1 (0.7%)
Bachelor	12 (92.3%)
Continuing education	
Master	4 (31%)
Specialist nurse	3 (23%)
Educational nurse	5 (38%)
Marriage	
Married	11 (85%)
Unmarried	2 (15%)
Years of intermediate professional title	
5∼10 years	10 (78%)
≥ 10 years	3 (22%)

### 3.4. Date Collection

In this study, semistructured one‐to‐one interviews and audio recordings were used to collect data. It began in December 2024 and ended in March 2025. After obtaining the consent, the informed consent was signed and carried out in a natural situation to create an equal, open, and heartfelt atmosphere without guidance and suggestion. Effective communication skills were fully used to encourage the subjects to describe their experiences and feelings about the promotion of senior titles. The interviewer made no comment on the content of the conversation, following the principle of suspension in qualitative research [27] and choosing open‐ended interview questions. The interview outline is as follows: (1) to assess the awareness of the promotion system (“do you know the senior title promotion system?”; “what do you think of it?”); (2) to capture lived experiences and emotions (“what are your thoughts and feelings about promotion?”; “how does it affect you?”); and (3) to explore readiness and coping (“are you ready for promotion?”; “how do you deal with it?”). Follow‐up on questionable parts by asking appropriate questions during the interview: for example, “can you tell me more about that?” “can you give me an example?” “tell me more?” wait for further confirmation of the problem. For instance, when a nurse being interviewed mentions “sometimes I feel lacking in energy,” the researcher would ask, “could you tell me more about it, or give an example?” The interviewee continued, “after work, I have to guide my children with their homework. By the time they finish their homework, it’s already past nine o’clock at night. I feel very tired and lack the energy to prepare for the promotion.”

Researcher Sufen Zheng is a registered nurse, she has participated in the relevant training in phenomenology research and has done qualitative research topics in phenomenology; she is responsible for data collection. Researcher Jiali Gong is a clinical nurse with a master’s degree. She has received training in phenomenology and has also conducted phenomenological research; she collaborated with researcher Sufen Zhang in data collection. Researcher Haixiang Zhu is a master’s degree supervisor; she has received training related to qualitative research and has been involved in the design and data analysis of the research. The interview was conducted in a quiet conference room of the hospital with no third person present, except the researcher and the interviewee. Before the interview began, there was a reminder sign hanging at the entrance: “The meeting room is in use. Please do not disturb,” to avoid the interview being interrupted. A written informed consent form was signed prior to the interview. Within 24 h after the interview, the speech was transcribed word for word into text, and the tone, pause, movement, and other information of the subjects were marked at that time. Each interview lasted 20–45 min. During the interview, in order to avoid bias, the researcher kept neutral and did not give any prompts, but gave some questions to explore deeper feelings and experiences. At the same time, the researchers do not express their emotions or thoughts, but rather position themselves as observers. Another registered nurse Jiali Gong, a graduate student (working at the same hospital), checked the recordings with the transferred transcripts to ensure the accuracy of the transcriptions. Data analysis was carried out separately for Sufen Zheng and Jiali Gong simultaneously. When there was no new topic (N11), two additional interviews were conducted (N12 and N13) until only the previous codes were repeated, indicating that the interview data had reached saturation, and the interviews were stopped.

### 3.5. Date Analysis

Sufen Zheng and Haixiang Zhu adopted the data analysis method of Colaizzi (1978) to analyze textual data. The reason for choosing this method is that Colaizzi’s data analysis method focuses on integrating the interviewee’s feelings, extracting phrases or sentences related to the studied phenomenon and adding steps for research participants to confirm and evaluate the researcher’s analysis conclusions. Moreover, it emphasizes the researcher’s suspension of previous views (that understanding does not depend on preunderstanding), which is very important in descriptive phenomenological research. The researchers did not bring in their own experiences and feelings. However, phenomenological analysis is more than just extracting text, requiring researchers to study the text in depth and gain insight into the phenomena being investigated [28]. Following Colaizzi’s seven steps, we read and examined each interview text to identify expressions that reflect the underlying meaning of the text as a whole. Line by line reading was done to extract phrases or sentences that are directly related to the experience and to identify salient issues from the narrative. This makes it possible to identify emerging themes. To illustrate our data analysis, we show in Table 2 how to identify important statements and how meaning formulation, thematic concentration, detailed description, and basic structure are described. In addition, all respondents were numbered 1–13. Based on the application of Colaizzi’s seven steps in phenomenological research [29] and previous studies [30], the generated themes were returned to the subjects for verification. The researcher inquired whether their true experiences had been captured in order to ensure the accuracy of the research results. N1, N6, and N12 were randomly selected to verify the analysis content of their text data, and three respondents agreed with the meaning extracted by the researchers without raising objections.

**TABLE 2:** The example of Colaizzi’s data analysis on the subtheme of “clear career development plan as soon as possible” in the theme of “Reflection”.

Excerpts of transcribed contents of the interview (what do you think of the professional title promotion system?)	Read repeatedly and identify significant statements	Formulate meanings	Cluster themes	Develop an exhaustive description	Produce the fundamental structure	Seek verification of the fundamental structure
N1: “The last few years have been a bit of a waste of time. Well, it’s just been clinical work, and uh (pause and thinking), the extra time without the preparation or whatever, or it would be different now.”	N1: “The last few years have been a bit of a waste of time. Well, it’s just been clinical work, and uh (pause and thinking), the extra time without the preparation or whatever, or it would be different now.”	*Code 1.* The lack of a clear goal and preparation led to time being somewhat wasted.	Early planning is essential, enabling proactive preparations.	After engaging in clinical practice for a certain period, the interviewed nurses expressed that had they been aware of the details of nursing promotion earlier, they could have initiated preparations sooner, such as writing articles. They could have commenced these preparations right after graduation, thereby avoiding the waste of time.	As soon as possible, formulate a clear career development plan (Reflection)	The generated topics were returned to the study respondents for verification. Ask if the researcher has captured their true experiences to ensure the accuracy of the results. If there is a deviation, the researcher must start from the first step and reanalyze it step by step.
N4: “Regret not having prepared earlier. Because I think if we’re right now, and there’s probably another one, there’s just so many things going on at home, and then you’re at work, and you’re really tied up.”	N4: “Regret not having prepared earlier. Because I think if we’re right now, and there’s probably another one, there’s just so many things going on at home, and then you’re at work, and you’re really tied up.”	*Code 2.* With advance planning, one could start preparing academic articles earlier.				
N11: “If I go back to the past, my supervisor may directly apply for graduate school after the exam, and I can rush it at that time. And then when it comes to writing articles, I think it’s too late.”	N11: “If I go back to the past, my supervisor may directly apply for graduate school after the exam, and I can rush it at that time. And then when it comes to writing articles, I think it’s too late.”					

### 3.6. Reflexivity and Bracketing

To minimize researcher bias, we adhered to phenomenological reduction (bracketing) [25] throughout the study. The interviewer (Sufen Zheng), a nursing manager, had no prior relationship with any participant. Before data collection, the research team held a bracketing session to explicitly identify and document personal preconceptions about nurse promotion (e.g., expectations that research pressure is the main barrier and assumptions about age‐related differences). These preconceptions were revisited during team analysis meetings to consciously set them aside. Additionally, peer debriefing was conducted: two researchers independently analyzed the first three transcripts and compared their findings, resolving discrepancies through discussion. Any disagreements about theme development were resolved by consensus among all authors. We also maintained an audit trail of analytical decisions to ensure transparency. Through these procedures, we aimed to ensure that the findings reflected participants’ lived experiences rather than researchers’ biases.

### 3.7. Ethics Considerations

This study has been approved by the hospital’s ethics committee, with the approval number 20240467. The principle of informed consent has also been followed [30]. Before the face‐to‐face interview, the researcher explained the purpose and content of the study to the participants, informed them that the entire interview process needed to be recorded and obtained a signed written informed consent form. During the interview, the interviewee had the right to withdraw without any consequences. At the end of the interview, each participant received a small thank you gift of no more than 15 yuan. All interviews were digitally encoded in N1∼13. Interview recordings and personal information were stored in an encrypted folder on the researcher’s computer. After the research report is completed, the recordings will be destroyed.

## 4. Results

Thirteen nurses (*n* = 13) were interviewed for this study, denoted by N1–N13. Age ranged from 34 to 45 years old, with an average age of 36.69 years old. All 13 nurses were in charge of nursing, and all had bachelor’s degree; two people graduated from an on‐the‐job graduate school and two people were studying in an on‐the‐job graduate school. There were 10 women and three men; two nurses were unmarried and had no children, the others were married and had children; three nurses were specialist nurses and five were educational nurses. The basic information of respondents is found in Table 1. This study identified five themes: (1) Escape; (2) Transform; (3) Challenge; (4) Reflection; and (5) Expect. These five themes formed a cyclical process: Escape drove Transform, which was repeatedly interrupted by Challenge, leading to Reflection, and culminating in Expect for systemic change.

### 4.1. Escape

In the interview, it was discovered that all the nurses were aware of the possibility of further promotion to senior titles after attaining intermediate titles. However, the majority opted to temporarily halt their pursuit of senior title promotion, refraining from taking further action. They held the view that the requirements for senior title promotion were extremely arduous and that their own capabilities were insufficient to meet these demands. This theme captures the initial motivational state of participants—a desire to retreat from the promotion track—which paradoxically served as both a barrier and a potential driver for subsequent change. According to the data, this theme consists of three subthemes: (1) perceived inadequacy; (2) clinical/family burden; and (3) lack of guidance/role models.

The respondents stated that they were aware of the requirements for promotion, but felt that their own abilities were insufficient to meet those requirements. This perceived inadequacy was not merely modesty but reflected a genuine gap between clinical training and research expectations.

N2: “*I feel that I want to write an SCI, like after we graduated from university, in fact, we have no contact, I am not a graduate student, we have no contact with systematic training, I feel that to write an article of that core, I feel too difficult.*”

N6: “*The project essay is too difficult…I can’t do it on my own.*”

N7: “*Well, I tried it, but it was still difficult, so I’ve given up now.*”

These statements reveal that the perceived inadequacy was not simply a lack of confidence but a realistic appraisal of skill gaps resulting from undergraduate education that did not emphasize research training.

The interviewed nurses also reported that the clinical work was busy and the free time after work was very limited, especially the married nurses with children needed more time to take care of their children. The second subtheme of this topic is clinical/family burden. This burden was disproportionately reported by female nurses with children, suggesting a gendered dimension to promotion challenges.

N3: “*The time after work in the evening is very limited, and I have to check my children’s homework. My husband is very busy. It can only say that after nine o’clock, he (the child) goes to bed, the rest of the time is mine. Rest time is also limited.*”

N7: “*After work back home, in fact, to tell the truth, because of the age also up, now is back, the obvious feeling is that year by year more and more powerless, I think I have anxiety ah. It’s also not efficient. Time passes quickly.*”

The sense of “powerlessness” expressed here connects Escape to Reflection: the inability to find time for promotion preparation forced nurses to reconsider whether the pursuit was worth the personal cost.

At the same time, the interview also showed that nurses felt the influence of the environment and the people around them was still great. The third subtheme of this topic is lack of guidance/role models. The absence of visible successful peers created an information vacuum, making the promotion pathway seem opaque and unattainable.

N6: “*Most of the colleagues around me rarely talk about the issue of promotion. I know that I can obtain a senior professional title, but I’m not sure what specific preparations are required.*”

N8: “*There is no one in the department (promoted senior title), in fact, I would like to know, for example, like me (want to promote the nurse). Then what kind of experience can be accumulated, including this process, certainly, he is familiar with it, can learn from everyone.*”

This lack of role models distinguishes intermediate nurses from junior nurses, who typically have clearer developmental pathways and observable supervisors to emulate.

### 4.2. Transform

The interview also showed that although nurses felt that it was difficult to be promoted to senior titles and it took more time, however, with the growth of work experiences, nurses’ views on promotion to senior titles were also changing. Transform represents the gradual identity shift from a “doer” (clinician) toward a “scholar‐clinician” who integrates research into practice. This topic included three subtopics: (1) clinical influence; (2) environmental impact; and (3) enhanced self‐awareness. Notably, Transform did not occur spontaneously; it was triggered by clinical demands and environmental exposure, suggesting that structured interventions could accelerate this shift. Nurses felt that scientific research and innovative thinking in clinical work can serve for clinical work.

N13: “*I think in this big environment, you have to learn something, you have to know something, you know nothing, you only know how to work, and now it is no longer possible, nurses can no longer only do work.*”

This statement reflects a growing recognition that clinical excellence alone is insufficient for career advancement in research hospitals.

N12: “*Last time, I shared a research case. I think it is very good. Learn from others, how to care for the stoma, what to do in the first step and what to do in the second step, we can use it immediately in the clinic.*”

At the same time, some nurses also felt that the help of people around them and the influence of the environment also changed their ideas about promotion. Environmental influence emerged as a critical catalyst: nurses working in research‐active departments were more likely to transform their professional identities.

N1: “*The scientific research and learning atmosphere of our department is very strong, and our department has a research team led by full-time graduate students, and there are activities every week. In the past two years, I have also spent more time on scientific research, including some topics or writing papers.*”

N5: “*I have colleagues around me who are preparing for promotion, and I think I can give it a try.*”

The contrast between this environment and the “lack of role models” described in Escape highlights how Transform is highly context‐dependent.

As nurses’ professional experience accumulates and their life experience broadens, their self‐awareness undergoes transformations. Enhanced self‐awareness was not merely about skill acquisition but involved a fundamental reorientation of professional identity and career aspirations.

N10: “*Working for a long time, I want to further improve myself, I want to be a head nurse. After that, last year, I started to enroll in this kind of part-time graduate school, um, and then I want to learn a little bit more about this system from this school.*”

N11: “*After reading about the specialist nurses, I felt compelled to do something. I noticed that many of the specialist nurses are excellent. And there are also many specialist nurses who are male, and they are all great, without any sign of giving up.*”

This suggests that exposure to successful peers—especially those with similar demographic characteristics—can effectively transform self‐perception and motivation.

### 4.3. Challenge

Data analysis revealed that nurses faced numerous challenges during the preparation for promotion to senior titles: (1) research learning; (2) degree hurdles; and (3) trial/failure. Critically, Challenge did not simply block progress; for some participants, it paradoxically triggered Reflection and reinforced Transform, while for others, it became a terminal barrier. This differential outcome depended largely on the availability of institutional support and peer encouragement.

Nurses reported that mastering scientific research knowledge posed significant difficulties. The majority of them admitted that during their undergraduate studies, they had not delved deeply into this area. These difficulties were not merely technical skill gaps; they reflected a structural mismatch between nursing education’s traditional clinical emphasis and the research‐intensive expectations of promotion systems.

N2: “*I feel like you can write an essay, it’s hard. We graduated from the university, we do not write papers, I am not a graduate student, we have no contact with systematic training, I feel to write a core of that kind of article, I feel too difficult.*”

This quote, similar to the one in Escape, appears again here to illustrate how the same initial difficulty can either trigger Escape (avoidance) or, when reframed, become a Challenge to be overcome through Transform.

N7: “*Statistics and scientific research methods…I don’t know. I don’t know where to start.*”

Nurses also perceive that they can strengthen their scientific research capabilities by upgrading their academic qualifications and professional competencies. Pursuing advanced degrees was seen as both a solution to research skill deficits and an additional burden, creating a double‐edged experience.

N10: “*Apply for graduate school…Systematic study and research methods. Since last year, I’ve been enrolling in this part-time graduate program, um, and I want to learn a little bit more about the system at this school.*”

N11: “*Consider graduate school…Make up for the lack of scientific research foundation. Went to part-time graduate school last year.*”

During the process of learning and submitting research articles, some nurses demonstrated that they were engaged in a cycle of repeated attempts and setbacks. The experience of failure was nearly universal, but its interpretation varied: nurses who framed failure as a learning opportunity (see also Reflection) were more likely to persist, while those who saw it as evidence of personal inadequacy were more likely to retreat into Escape.

N2: “*I wrote the article a few times…All rejected, anxious.*”

This nurse’s anxiety reflects the emotional toll of repeated failure without structured support.

N10: “*The article was revised repeatedly and finally accepted, I think it is important to insist. In fact, through this study last year, I also wrote an article in my own words, anyway, it is more tortuous, invested in several journals, and the last word is already hired, before and after about a year.*”

N10’s eventual success, achieved through persistence, illustrates how Challenge can be overcome—but the year‐long timeline also reveals the substantial time cost that many nurses cannot afford given their clinical and family responsibilities.

### 4.4. Reflection

In the interview process, it was also found that most nurses had more or less reflections on the promotion of senior titles, including (1) early career planning; (2) perseverance; and (3) continuous self‐growth. Reflection emerged as a cognitive coping strategy that allowed nurses to reinterpret setbacks, adjust strategies, and maintain motivation despite repeated challenges. It often acted as a bridge between Challenge and Expect.

The interviewed nurse stated that it is necessary to clearly define one’s career development plan as early as possible, including specific goals and action plans. The regret expressed about “wasted time” suggests that early career planning is a modifiable factor that hospitals could address through structured orientation and mentorship programs.

N1: “*The last few years have been a bit of a waste of time. Well, it’s just been clinical work, and, uh, the extra time without the preparation or whatever, or it would be different now.*”

N13: “*If in college, uh, knowing that I was still in the clinic for so long, it was really, uh, just personal career planning, that would definitely change.*”

In the process of preparation, the nurse also said that the spirit of perseverance is needed. Perseverance was not simply a personality trait; it was often described as a deliberate choice made in response to external pressures or examples set by peers.

N5: “*Try even if it is difficult…If you don’t try, you will fail. I had to. I had to do it. It was real. I had to do it. That’s it. I might have been lazy before.*”

The phrase “I had to do it” suggests that perseverance was sometimes driven by external expectations or a fear of stagnation rather than intrinsic motivation.

N8: “*Project failed…Keep improving and don’t give up. Whatever it is, give it a try first. Well, you’ve been to all the lectures, so see if you can do it, and then you can do it.*”

Nurses also said that with the changes in positions and perceptions, their goals for themselves are also constantly improving. Continuous self‐growth represents an upward spiral: successful experiences (e.g., completing a project and gaining a new role) generated increased self‐efficacy, which in turn motivated further growth. This finding challenges the assumption that promotion barriers inevitably lead to burnout; for some nurses, barriers can fuel growth.

N7: “*Educating nurses and pushing me to learn management.*”

N11: “*When the research was done, it was very interesting. I think it is very difficult to write and do at the beginning, but I think it is quite interesting. Love what you do, and in the last two or three years, I want to improve myself (professionally).*”

N11’s journey from finding research “difficult” to “interesting” exemplifies the transformative potential of sustained engagement with Challenge, supported by Reflection.

### 4.5. Expect

In the interview process, the interviewed nurses also expressed their expectations: (1) career/psychological support; (2) research environment; and (3) diversified criteria. Expect represents the culmination of the cyclical process: after experiencing Escape, undergoing Transform, confronting Challenge, and engaging in Reflection, nurses articulated concrete hopes for systemic change. These expectations were not abstract wishes but practical recommendations grounded in their lived struggles.

N1: “*I hope junior nurses can know the promotion requirements early.*”

This expectation directly addresses the “lack of early career planning” identified in Reflection, suggesting a policy solution: transparent, early dissemination of promotion criteria.

N2: “*It is for those of us who have not written that kind of articles, feel the need for a leader, on the system to train.*”

The call for “a leader” and “system to train” reflects a desire for structured mentorship—a direct response to the “lack of guidance” subtheme in Escape.

N6: “*I hope the department has a team to learn to write articles together. Some departments have a very good atmosphere for scientific research, feeling that everyone should participate, and everyone can’t lie flat in this department.*”

The mention of “lie flat” (tang ping)—a contemporary Chinese term for disengaging from competitive pressures—reveals that without institutional support, some nurses may opt out of the promotion track entirely.

N8: “*Clinical nurses must be clinical, is there any other form can replace this article? Like substituting clinical results for scientific research.*”

This expectation directly challenges the current research‐centric criteria, aligning with national policy rhetoric [11] but highlighting the gap between policy intention and institutional implementation.

## 5. Discussion

Through an in‐depth analysis of the experiences of mid‐level nurses during their promotion to senior titles, this study has identified five overarching themes and 15 subthemes, namely “Escape”, “Transform”, “Challenge”, “Reflection”, and “Expectation”. Previous studies [31] have identified four main themes regarding nurses’ thoughts on career development: helping others through a good job; various motivations for self‐improvement; lack of autonomy and restricted decision‐making as obstacles; and challenges in time planning and allocation. Among them, various facilitating factors that promote self‐development and time management challenges are similar to the two subthemes of this study: being overwhelmed by clinical work and family responsibilities and self‐motivation and enhanced self‐awareness. Furthermore, after Transform topic, our study identified some new themes: Challenge, Reflection, and Expectation, which were not discovered in previous studies. This divergence likely reflects the unique pressure of China’s research‐oriented promotion system, which prioritizes publications—a demand less emphasized in the contexts studied by [31]. This might be related to the promotion system in China, especially as promotions are closely tied to clinical work and research achievements [14]. In research‐oriented hospitals, the majority of nurses hold a bachelor’s degree. But they received limited research training in school, so they need to face more learning and challenges [32]. The evaluation systems for nurse promotions in other countries adopt the hierarchical advancement model, but the implementation of this model varies from country to country [33]. Among them, the nurses are more concerned about career development opportunities, fair treatment, and salary packages [34]. These research outcomes not only unveil the intricate psychological states and behavioral patterns of nurses throughout the promotion process but also furnish a crucial foundation for the optimization of the nursing career development system.

The study revealed that the three subthemes subsumed under the “Escape” theme—perceived inadequacy, clinical/family burden, and lack of guidance/role models—vividly encapsulated nurses’ initial reactions to the pressures of promotion. This finding aligns with the research by Sun et al. [12] on burnout among senior nurses. However, while Sun focused on burnout as an outcome, our study reveals that Escape is often a strategic pause rather than pure burnout—some nurses explicitly framed it as “waiting for better timing.” This study also found that unmarried and childless nurses also expressed that their self‐study time after work was limited. This contradicts the assumption that only nurses with family responsibilities face time constraints, suggesting that clinical workload—not family status—may be the primary barrier. Whether this is related to their personal schedule or the busyness of their clinical work still requires further research. Notably, this avoidance mentality is prone to gradual transformation influenced by the factors elucidated in the “Transform” theme, including the impact of clinical practice, the influence of the professional environment and peers, the pursuit of self‐improvement, and the enhancement of self‐awareness. As posited by Shao et al. [35], a conducive practice environment and positive professional identity can significantly facilitate the career development of nurses. Within the “Change” theme, the infection of the environment and the people around it is of particular concern. The study found that positive team atmosphere and role model power can significantly improve nurses’ promotion intention [17]. This suggests that hospital managers should attach importance to creating a supportive organizational culture, which is consistent with the findings of Zheng et al. [30] on the effect of team support and systematic training. Our study extends Zheng et al.’s work by demonstrating that team support is most effective during the Transform stage but insufficient during the Challenge stage without additional methodological support. This journey from Escape to Transform reflects the mechanism of self‐growth in the career advancement of nurses. This is in line with the psychological theory of human self‐regulation and adaptation [36]. At the same time, three of the interviewees in this study stated that after making efforts, they realized that their current abilities were insufficient to achieve the goal of advancing to a higher professional title and thus they decided to give up the promotion. This is also a phenomenon that was not discovered in previous studies. This finding challenges the implicit assumption that effort inevitably leads to promotion; it suggests that accurate self‐assessment can lead to rational withdrawal, which may be a legitimate career decision rather than failure.

In the “Challenge” theme, subthemes such as the learning of scientific research knowledge, the improvement of self‐education, and continuous trial and failure highlight the main difficulties of nurses in the promotion process. These findings are highly consistent with the policy requirements of the Zhejiang Provincial Health Commission [4] and explain why many nurses view research pressure as the biggest barrier to promotion [5]. However, our findings reveal a critical policy‐practice gap: policies assume research readiness, but nurses report lacking even basic training in statistics and scientific writing—a mismatch that previous studies have not directly documented. On the other hand, this also indicates that for clinical nurses, the research indicators stipulated in the policies are challenging. Without continuous learning and improvement, it is difficult to meet these requirements. This is precisely what policy makers need to consider: how can policies be made to better meet clinical needs while also exceeding them? Of particular concern, nurses who were able to overcome these challenges often displayed the qualities mentioned in the “Reflection” theme: early clarity on career development, perseverance, and a constant goal of self‐growth. This is in line with the research conclusion of Huang et al. [21] on the importance of career planning. Unlike Huang et al.’s survey‐based study, our phenomenological data show that early planning alone is insufficient; it must be accompanied by sustained institutional support during the Reflection stage. Among them, a male nurse mentioned that over the past year, he had spent almost all his time in the process of submitting articles, revising them, and receiving rejections. However, he also stated that he would continue to persevere. This might also be related to the characteristics of nurses, such as their diligence, reliability, and hard work.

The “expect” theme found in the study (career/psychological support, research environment, and diversified criteria) provides a clear direction for hospital management. These expectations align with the previous emphasis on effective guidance and the recognition of the importance of career path planning [2, 30]. It is important to note that these expectations of nurses are not one‐way requests, but are based on self‐improvement embodied in the themes of “Change” and “Reflection”. The subtheme of “Expectation” to provide diversified promotion assessment standards directly points to the shortcomings of the current professional title evaluation system. The study found a clear disconnect between a single research‐oriented evaluation criterion and the diverse career development needs of nurses [16]. This requires policymakers to make reference to international experience (such as the APRN training model proposed by Kerr and MacAskill [10]) and establish a more flexible evaluation system. However, direct importation of Western competency‐based models may be insufficient without addressing China’s unique context—particularly the deeply embedded expectation of publication output in research hospitals. At the same time, the subtheme of self‐education promotion in the theme of “Challenge” reflects the inherent needs of nurses for professional development. This echoes the findings of Cai et al. on the needs of nurses’ career development [22]. Cai et al. studied nurses in general hospitals; our study extends this by showing that in research hospitals, the need for research training is not merely a “preference” but a prerequisite for promotion eligibility.

However, the results of this study support the conclusions of other researchers, namely, that it is more difficult for nurses to be promoted to senior positions compared to doctors [37]. In the future, more attention should be paid to the individual needs of nurses, in order to establish an appropriate training system and make plans in advance.

## 6. Limitation

Using phenomenological method, this study only discusses the feelings and experiences of nurses with intermediate titles on promotion to senior titles. This study did not select personnel who had already achieved promotion to senior professional titles for the interviews. Other factors related to these experiences are not discussed and mentioned. In addition, this study was conducted in one hospital, and the selection of interviewees had certain limitations. We also failed to recruit more male nurses and more nurses with MSN. Whether the researcher’s relationship structure would have any impact has not been taken into consideration. Future research could consider the pitfalls mentioned above to find out more about nurses’ life experiences of promotion.

## 7. Conclusion

The promotion of nurses represents a pivotal stage in their career development. This research revealed that intermediate‐level nurses underwent psychological transformations and self‐capacity enhancement during the process of advancing to senior titles, with both negative and positive experiences. Unlike previous studies that focused on promotion barriers alone, this study’s unique contribution is the identification of a cyclical process—Escape ⟶ Transform ⟶ Challenge ⟶ Reflection ⟶ Expect—demonstrating how intermediate nurses actively navigate, reinterpret, and sometimes transform obstacles into growth opportunities.

Theoretically, these findings advance nursing management theory by challenging deficit‐based assumptions about promotion barriers. The cyclical model reveals that Challenge does not merely block progress but can paradoxically trigger Reflection and reinforce Transform when institutional support is present. This extends career plateau theory [15] by showing that plateauing is not simply a function of time but of policy–practice misalignment.

Based on the research results, it is recommended that nursing managers should prioritize the early‐stage career planning of nurses. They should endeavor to cultivate an environment conducive to scientific research and innovation, thereby fostering nurses’ research capabilities. Additionally, nursing managers ought to offer comprehensive support and systematic training to nurses throughout the promotion process. Such measures can effectively alleviate the pressures and difficulties faced by nurses and facilitate their professional advancement.

The outcomes of this study hold significant value for nursing administrators, clinical educators, and academic nursing educators. By understanding the cyclical, emotionally complex promotion experiences of intermediate nurses, managers can move from generic support to targeted, theme‐based interventions that enhance both promotion success rates and professional identity.

## 8. Implications for Nursing Policy

Based on our findings, we propose the following strategies: for nurse managers: (1) establish structured mentorship programs pairing intermediate with senior nurses; (2) arrange dedicated research time based on the actual situation; (3) provide on‐site methodological support (e.g., statistics and writing help); and (4) offer annual career planning workshops. For policymakers: (1) diversify promotion criteria to include clinical projects, teaching, and certifications alongside publications; (2) create innovation‐friendly environments (seed grants, journal clubs, and recognition awards); and (3) integrate career development and research methods into nursing school curricula.

These strategies directly address the five themes identified in our study: Escape (mentorship and protected time), Transform (innovation environment), Challenge (methodological support), Reflection (career planning workshops), and Expect (diversified criteria). Adjustments should be made according to local contexts.

## Author Contributions

Study design: Sufen Zheng and Haixiang Zhu; data collection: Sufen Zheng and Jiali Gong; data analysis: Sufen Zheng, Haixiang Zhu, and Jiali Gong; methodology: Sufen Zheng; drafting the manuscript: Sufen Zheng; writing, editing, and supervision: Sufen Zheng and Haixiang Zhu.

## Funding

This work was supported by the Medical Health Science and Technology Project of Zhejiang Provincial Health Commission (grant number 2023KY784) and the Project of Teaching Reform of the Third Medical College of Zhejiang University (grant number A‐16).

## Disclosure

All authors reviewed and approved the submitted version.

## Ethics Statement

This study has been reviewed by the Ethics Committee of Sir Run Run Shaw Hospital and follows the informed consent principle.

## Conflicts of Interest

The authors declare no conflicts of interest.

## Data Availability

The data that support the findings of this study are available from the corresponding author upon reasonable request.
